# Prognostic Disclosure in Metastatic Breast Cancer: Protocol for a Scoping Review

**DOI:** 10.2196/57256

**Published:** 2025-04-29

**Authors:** Linda Battistuzzi, Irene Giannubilo, Claudia Bighin

**Affiliations:** 1 Unit of Medical Oncology 2 IRCCS Ospedale Policlinico San Martino Genoa Italy

**Keywords:** scoping review, protocol, metastatic breast cancer, advanced breast cancer, prognostic disclosure, prognosis, communication, truth-telling, ethics, shared decision-making

## Abstract

**Background:**

Prognostic disclosure enables shared decision-making by keeping patients informed about their health and involved in treatment-related choices. In metastatic cancer, patients who understand their prognosis are better placed to evaluate their health situation, have more realistic expectations about treatment, and better end-of-life (EoL) care. However, many oncologists feel uncomfortable discussing prognoses with their patients, as they worry about causing psychological distress and diminishing hope. Other barriers to prognostic disclosure are associated with the inherent uncertainty of prognostication, which is especially prominent in metastatic breast cancer (mBC), as are a lack of timely discussions about EoL care and poor quality of care at EoL. Despite this background, a preliminary literature search has shown that, to date, knowledge about prognostic disclosure in mBC has not been systematically mapped.

**Objective:**

The overall aim of this scoping review will be to comprehensively explore the existing literature pertaining to prognostic disclosure in mBC.

**Methods:**

This scoping review will follow Arksey and O’Malley’s expanded framework. We will systematically search electronic databases for peer-reviewed, published journal articles to identify appropriate studies. First, 2 members of the research team will independently review titles and abstracts. Then, they will review full texts to establish whether articles meet inclusion criteria. A data chart for collecting and sorting information will be developed. Results will be reported following the PRISMA-ScR (Preferred Reporting Items for Systematic Reviews and Meta-Analyses extension for Scoping Reviews) guidelines and summarized quantitatively and qualitatively.

**Results:**

We expect to present the results in a scoping review in 2025.

**Conclusions:**

To our knowledge, this scoping review will be the first systematic effort aimed at mapping existing literature on prognostic disclosure in mBC, in which rapidly developing therapeutic approaches and changing disease trajectories generate new uncertainties and place new communicative demands on oncologists. The insights gained from this scoping review will inform the development of an interview schedule with oncologists, oncology residents, oncology nurses, and patients with mBC to explore their experiences, views, and perceptions about prognostic disclosure.

**International Registered Report Identifier (IRRID):**

PRR1-10.2196/57256

## Introduction

### Background

Metastatic breast cancer (mBC), also termed advanced or secondary breast cancer, occurs when the primary lesion spreads to other sites of the body, most commonly the bones, lungs, brain, or liver. In recent years, new targeted systemic therapies have improved the median overall survival of many patients with mBC, and some patients may be cured by combination therapy or have long-term responses. However, resistance to treatment remains a significant challenge, so most patients will eventually progress and die from their disease [1].

Prognostic disclosure—communicating the likelihood that a certain clinical state or outcome will occur within a certain period—is now a routine part of cancer care in many countries [2]. Grounded in the ethical principles of autonomy and self-determination [3], prognostic disclosure enables shared decision-making by keeping patients informed about their health and involved in treatment-related choices, especially in situations of uncertainty. Patients who understand their prognosis tend to have more realistic expectations about treatment [4]; they are also better able to prepare for the future, have higher quality end-of-life (EoL) care and have better chances of dying in their preferred place [5]. Conversely, concealing a poor prognosis interferes with patients’ autonomy and ability to make treatment-related decisions that are aligned with their values, preferences, and goals, often resulting in the pursuit of futile therapies and poor symptom management [3,6].

To promote the value of prognostic disclosure and help clinicians become more familiar with it, consensus guidelines specifically addressing patient-clinician communication have been developed [7]. Yet, many oncologists continue to feel uncomfortable communicating prognostic estimates to their patients, often due to concerns about causing psychological harm and diminishing hope [8-10].

Research on prognostic awareness and its association with anxiety, depression, and quality of life (QoL) among patients with metastatic cancer supports these concerns to some extent, as the evidence is not uniform. Some studies report that prognostic awareness is associated with low anxiety, low depression, and high QoL [11], while others report a varied range of outcomes, including negative psychological impacts [12]. This may reflect the fact that prognostic disclosure that successfully balances hope and realism requires skillful, empathic communication [12-14].

Apart from these possible harms, other barriers to prognostic disclosure have been identified. Prognostication is inherently uncertain, owing to the randomness of future outcomes and the lack of evidence on the probability of outcomes. This lack of evidence is increasingly prominent in oncology because of the rapid pace of advancements in personalized medicine and novel therapies and techniques [15]. In mBC, reducing prognostic uncertainty is especially complex, as the clinical behavior of mBC varies greatly, and the disease trajectory is difficult to predict [16-18]. In addition, studies have found that timely discussions about EoL care, documentation of patient preferences in health records, and quality of care at EoL are particularly lacking for patients with mBC [19]. Thus, it is perhaps not surprising that this patient population has been found to be at increased risk of being overtreated at EoL [20,21], with insufficient access to hospice services and often dying in acute care environments [19].

Despite this landscape, the increasing incidence of mBC, and the large number of patients living with the disease, a preliminary literature search has shown that, to date, knowledge about prognostic disclosure in mBC has not been systematically mapped.

### Study Objectives

The overall aim of the proposed scoping review is to identify, summarize, and synthesize knowledge about prognostic disclosure in mBC. We expect that the results of this work will facilitate further research aimed at gathering evidence and gaining insights on the process and outcomes of prognostic disclosure in mBC to help guide medical professionals in challenging yet crucial conversations with patients.

## Methods

### Study Design

This scoping review will be conducted following the widely known 5-stage framework by Arksey and O’Malley [22] and subsequent recommendations [23,24]. The results will be reported in accordance with the PRISMA-ScR (Preferred Reporting Items for Systematic Reviews and Meta-Analyses extension for Scoping Reviews) guidelines.

A scoping review is a rigorous, transparent tool that can be used to map or synthesize a range of evidence in order to convey the size and scope of a research field [24]. According to Arksey and O’Malley [22], by conducting a scoping study researchers can survey the extent, range, and nature of research activity in a given field, establish whether a full systematic review is warranted, summarize and disseminate research evidence, or identify gaps in the existing literature.

Compared to systematic reviews, which aim to summarize the best available research related to a given research question, scoping studies generally do not critically evaluate the quality of the studies they cover [25,26]. Compared to narrative or literature reviews, scoping reviews require analytical reinterpretation of the literature. Scoping reviews are helpful when evidence on a topic is emerging or the relevant literature is complex or heterogeneous. In addition, they can cover findings from a range of different study designs and methods [24]. Thus, they are well-suited to synthesizing the literature on a topic that has yet to be comprehensively mapped, such as the one we will endeavor to review.

### Stage 1: Identifying the Research Questions

The research questions of this scoping review are the following: What has been reported about prognostic disclosure in mBC? What are the knowledge gaps regarding prognostic disclosure in mBC?

An exploratory search in PROSPERO—the International Prospective Register of Systematic Reviews of the National Institute for Health Research—and the Cochrane Library revealed no comprehensive systematic reviews addressing similar questions. The research team will revise the protocol as appropriate during the review process.

### Stage 2: Identifying Relevant Studies

#### Eligibility Criteria

To decide the eligibility criteria, the research team followed the PCC (Population/Concept/Context) framework for scoping reviews by the Joanna Briggs Institute [27]. Inclusion criteria are (1) studies wherein the population comprises individuals older than 18 years with mBC and health care professionals (oncologists, oncology residents, and oncology nurses) providing clinical care to patients with mBC, (2) the concept is prognostic disclosure by health care professionals to patients with mBC, and (3) the context is a health care setting.

The following items will comprise further inclusion criteria: articles published in a peer-reviewed outlet, articles in English, articles that include the relevant search terms (see below), articles in which prognostic disclosure to patients with mBC is the main focus or is at least addressed in its own part or section, and articles that were published in 2010 or later, in light of the changes that have since taken place in mBC treatment.

Our exclusion criteria are as follows: articles that focus on prognostic disclosure but only marginally mention mBC and articles that focus on mBC but only marginally mention prognostic disclosure.

#### Search Strategy and Information Sources

Literature search strategies will be developed using MeSH (Medical Subject Headings) terms and text words related to prognostic disclosure in mBC. Studies will be identified by searching MEDLINE, CINAHL, Embase, and PsycInfo. Targeted internet searching using Google Scholar will also be performed to identify additional studies of interest. A hand search of the reference lists of eligible articles will be carried out to ensure literature saturation. The search strategy will be reviewed by a specialist librarian using the “Peer review of electronic search strategies” guidelines [28].

### Stage 3: Selecting the Studies

Results retrieved from all databases will be imported into a reference management software, and duplicate references will be removed. At the first screening stage, 2 researchers will independently review the title and abstract of all references and assess them for eligibility. Disagreements will be discussed until consensus is reached, and if necessary, by consulting a third independent researcher. Articles not related to the topic, theses, articles from the popular press, reports, nonreviewed books and book chapters, presentations, and opinion pieces will be excluded. The 2 researchers will then independently conduct the second eligibility screening, evaluating full-text articles against the inclusion and exclusion criteria. Results will be compared, and any disagreements will again be discussed to reach a consensus on final inclusion, involving the third researcher if needed. A PRISMA-ScR flowchart will be developed to report the process.

### Stage 4: Charting the Data

Based on the recommendations by Levac et al [24], we will perform a descriptive quantitative synthesis and a thematic analysis. In line with Arksey and O’Malley [22], our descriptive quantitative summary will begin with the final number of articles included in the scoping review and the essential characteristics of the articles identified, such as first author, year of publication, country, discipline/field of inquiry, study design (ie, qualitative, quantitative, mixed methods, retrospective, prospective, longitudinal, and cohort study), research methods, type of health professional and/or patient involved, number of participants, etc. This information will be presented in the scoping review report to provide an overview of the included literature.

For qualitative data, content analysis will describe the data extracted, highlighting consistencies and differences [29]. The articles will be coded following a multistep process, including open coding, axial coding, and selective coding, using the Dedoose web-based application [30]. In the first phase, units of meaning will be identified and labeled to allow categories to emerge from the data (open coding). Open codes will be categorized, with similar codes grouped, refined, and combined into larger themes (axial coding). The conceptually stable thematic patterns that emerge will then be organized and grouped into higher-order conceptual themes. Selective coding will involve integrating and refining of these concepts [29]. Finally, findings will be integrated and validated through discussion among all members of the research team.

### Stage 5: Collating, Summarizing, and Reporting the Results

The essential characteristics of the studies will be tabulated, providing an overall summary of the articles selected. Other results will be presented in diagrams and descriptive summaries, as most appropriate to display and convey the extracted information.

### Ethical Considerations

The scoping review methodology consists of reviewing material that is publicly available and does not involve any patient-related data, so this study is not subject to ethical approval.

## Results

We expect to present the results of this work in a scoping review in 2025.

## Discussion

Patients with a limited life expectancy, such as those with mBC, need information about their prognosis to make sense of their health-related situation, participate in decisions about their treatment, and prepare for the future [31]. However, while information provision is one of the cornerstones of shared decision-making [32], prognostic disclosure can lead to negative effects on patients’ well-being, eliciting anxiety, hopelessness, and demoralization [33,34]. Oncologists thus need to find a balance between conveying clear information but not overwhelming patients, being realistic and simultaneously remaining hopeful [12-14], which may leave them feeling torn [35].

To our knowledge, this scoping review will be the first systematic effort aimed at mapping existing literature on prognostic disclosure in mBC. We expect to find that evolving disease trajectories, increasing survivorship, and the need for decision-making about treatment options in the context of rapidly developing therapeutic approaches all place significant new communicative demands on oncologists navigating the challenges and complexities of prognostic discussions [35].

The results of this work will be used to inform the development of an interview schedule with patients with mBC, oncologists, oncology residents, and oncology nurses to explore their experiences, views, and perceptions about prognostic disclosure in mBC. Our findings will be disseminated through presentations at conferences and webinars.

## Abbreviations

EoL: end of life
mBC: metastatic breast cancer
MeSH: Medical Subject Headings
PCC: Population/Concept/Context
PRISMA-ScR: Preferred Reporting Items for Systematic Reviews and Meta-Analyses extension for Scoping Reviews
QoL: quality of life
